# The role of helium gas in medicine

**DOI:** 10.1186/2045-9912-3-18

**Published:** 2013-08-04

**Authors:** Carlos J Berganza, John H Zhang

**Affiliations:** 1 Departments of Neurosurgery and Physiology, Loma Linda University, Loma Linda California, USA

**Keywords:** Helium, Heliox, Inhalation therapy, Cardioprotection, Neuroprotection, Insufflation

## Abstract

The noble gas helium has many applications owing to its distinct physical and chemical characteristics, namely: its low density, low solubility, and high thermal conductivity. Chiefly, the abundance of studies in medicine relating to helium are concentrated in its possibility of being used as an adjunct therapy in a number of respiratory ailments such as asthma exacerbation, COPD, ARDS, croup, and bronchiolitis. Helium gas, once believed to be biologically inert, has been recently shown to be beneficial in protecting the myocardium from ischemia by various mechanisms. Though neuroprotection of brain tissue has been documented, the mechanism by which it does so has yet to be made clear. Surgeons are exploring using helium instead of carbon dioxide to insufflate the abdomen of patients undergoing laparoscopic abdominal procedures due to its superiority in preventing respiratory acidosis in patients with comorbid conditions that cause carbon dioxide retention. Newly discovered applications in Pulmonary MRI radiology and imaging of organs in very fine detail using Helium Ion Microscopy has opened exciting new possibilities for the use of helium gas in technologically advanced fields of medicine.

## Introduction

Helium is a very light, odorless, tasteless, and colorless noble gas with a strong safety profile and multiple applications. It does not support combustion. It is unique in that the boiling point −452.1°F (−268.9°C) and melting points −458°F (−272.2°C) are the lowest among the elements. Helium is the second most abundant element in the universe. Despite this, most of the helium in use is a byproduct of radioactive decay underground and must be extracted during natural gas production. Helium is used for purposes that require some of its unique properties: its low density, low solubility, and high thermal conductivity. 7,000 tons, or 22%, of the total helium used involves the cooling of superconducting magnets in medical magnetic resonance imaging (MRI) scanners. Though commonly known as a lifting gas in balloons and airships, this use accounts for less than 7% of the total used [1].

**Figure 1:**
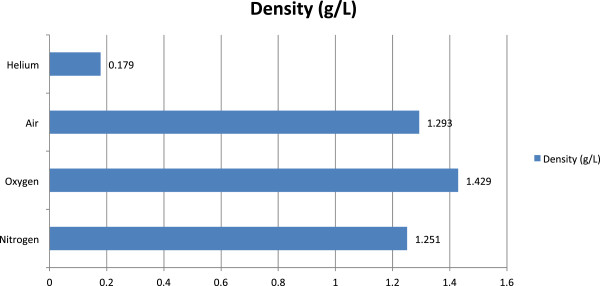
The density of helium is substantially lower than other gases, accounting for its multiple applications in respiratory therapy.

Helium is the lightest noble gas (4 g/mol). The only gas with a lower density than helium is hydrogen [2]. The use of hydrogen is more limited than helium because of its flammability in air mixtures. Helium (0.179 g/L) is 86% less dense than room air (1.293 g/L) and 8 times less dense than oxygen (1.429 g/L) (Figure 1). This unique property has been critical to its multiple applications.

The high thermal conductivity of helium results in lower body temperature when the body is embedded in helium, which could result in decreased metabolism and decreased energy expenditure [3]. Hypothermia was induced in rats breathing 75% helium for extended periods [4]. In humans, breathing helium for short periods has not been shown to induce hypothermia [5].

## History of helium as a therapeutic gas

In 1926, Sayers and Yant found that helium-oxygen mixtures could be breathed by humans without discomfort, and by animals without demonstrable ill effects. Due to the lower solubility of helium compared with nitrogen, using a mixture of helium and oxygen (Heliox) rather than nitrogen and oxygen decreased the formation of nitrogen bubbles and therefore decompression illness in deep-sea divers [6].

In 1934, Barach was first to propose using Heliox as a therapeutic gas [7]. Since a helium/oxygen mixture (79/21) has a weight that is one-third compared with air, Barach proposed using this lighter gas to improve the flow of oxygen in patients with upper airway obstruction and asthma exacerbation [8-10].

**Figure 2:**
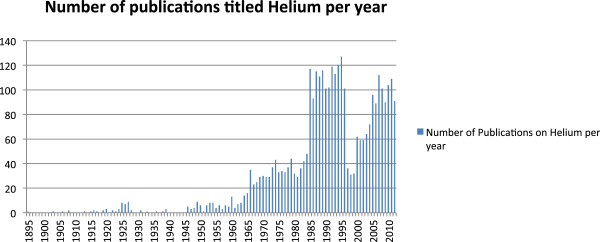
**A search of PubMed for articles titled helium shows an increased number of publications with an interest in helium within the last 10 years.** A PubMed search was performed with helium contained in the text of the title and the search results were counted and graphed by year.

Interest in the clinical use of helium declined temporarily after this period of time due to the advent of bronchodilators and mucolytic agents and also because of the loss of sources of helium production during the Second World War [11]. In the 1980s, interest in helium gas research resurfaced as the number of asthma related deaths began to rise [12]. Within the last 10 years, there has been an increased interest in studies involving the use of Helium (Figure 2).

Unlike the noble gas xenon, helium does not have anesthetic properties [13]. Given in higher pressures predicted to be anesthetic by the Meyer-Overton hypothesis, helium actually increases the minimum alveolar concentration for volatile anesthetics and also is shown to produce convulsions. Helium is therefore characterized as a non-immobilizer, a gas that does not induce anesthesia, but may still have other biological effects [14].

## Effects of helium on different organs

### Helium and the respiratory system

The flow of a gas depends on the density and viscosity of each element within a gas mixture. The low density of helium reduces airway resistance and promotes airflow through the lungs. Heliox decreases the work of breathing (WOB) in patients with increased airway resistance [15]. Therefore heliox has been reported to be effective in a variety of respiratory conditions including upper airway obstruction, asthma exacerbation, post-extubation stridor, croup, bronchiolitis, chronic obstructive pulmonary disease (COPD), acute respiratory distress syndrome (ARDS), and pulmonary function testing [16-21]. In a rat model simulating ARDS, heliox was effective in decreasing neutrophil infiltration, interstitial/intraalveolar edema, perivascular and/or intraalveolar hemorrhage, and hyaline membrane formation histopathologically [22].

**Figure 3:**
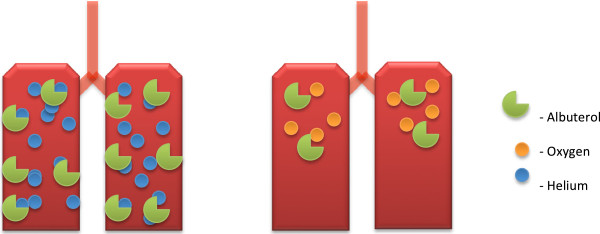
Because of the very low density of helium, there is better delivery of albuterol nebulized with helium (Left) compared with albuterol nebulized with oxygen (Right) in the lungs.

In 1938 Sykes and Lawrence found that an artificial atmosphere of helium and oxygen is practically about twice as easy to breathe as ordinary air [23]. In stable asthmatics, is has been shown that Heliox led to more effective deposition of radiolabeled particles in the lung [24]. During severe acute asthma exacerbations, it has been found that albuterol nebulized with Heliox leads to a more significant improvement in spirometry when compared with albuterol nebulized with oxygen, because the low-density gas improves albuterol deposition in the distal airways (Figure 3) [25]. In infants with moderate-to-severe respiratory syncytial virus (RSV) bronchiolitis, Heliox therapy improved their clinical scores and reduced accompanying tachycardia and tachypnea [26]. Heliox also improves the elimination of CO_2_. Compared with a mixture of N_2_/O_2_, CO_2_ diffuses four to five times faster in Heliox. This property significantly reduces hypercapnia and normalizes pH [27].

Breathing Heliox increases the duration of exercise training that can be performed in patients with chronic obstructive pulmonary disease (COPD), augmenting the known benefits of a comprehensive pulmonary rehabilitation program [28]. In acute status asthmaticus in children, inhalational Heliox therapy significantly improved pulsus paradoxus, peak flow, and dyspnea. Heliox also reduced the work of breathing in children, with the potential to prevent respiratory muscle fatigue and ventilatory failure [29].

### Helium and the cardiovascular system

It has been shown that myocardial tissue can be protected against ischemia by subjecting it to one or a few short ischemic episodes before ischemia. Early preconditioning (EPC) or late preconditioning (LPC) before ischemia or post-conditioning (Post C) after myocardial ischemia, ameliorates myocardial ischemia [30]. In a study on myocardial infarct size in rabbits, three 5-minute cycles of 70% helium, neon, and argon modestly reduced myocardial infarct size [31].

It has also been shown that helium inhalation enhances vasodilator effect of inhaled nitric oxide on pulmonary vessels in hypoxic dogs. Compared with nitric oxide (NO) in nitrogen gas (N_2_), the mean pulmonary artery pressure decreased significantly after NO inhalation in helium, indicating a greater vasodilator effect of NO in helium. It is likely that NO delivery to the periphery of the lungs was facilitated by helium more than by N_2_[32].

In a study of 17 healthy men subjected to 15 minutes of submaximal exercise on an Elema-Schonander cycle ergometer while breathing ambient air or Heliox, it was shown that Heliox improved exercise tolerance while reducing rising lactate and pyruvate levels compared with ambient air [33].

Helium has been demonstrated to protect myocardial tissue from ischemia by various different mechanisms. Pagel et. al performed a left-anterior descending (LAD) coronary artery occlusion in rabbits and found that animals treated with a helium gas mixture had significantly reduced infarct size compared with control groups. They found that the cardioprotection was due to the activation of pro-survival signaling kinases and the inhibition of mitochondrial permeability transition pore (mPTP) opening [31]. Later, they found that Helium maintains intracellular acidosis during early reperfusion, which inhibits mitochondrial transition pore formation [34]. In subsequent studies, researchers found that reactive oxygen species (including nitric oxide) [35,36], mitochondrial adenosine triphosphate-regulated potassium channels, [37], and opioid receptors [38] were responsible for the cardio-protective effects induced by helium preconditioning.

Heinen et. al applied Heliox as a pre-conditioning modality in a rat heart ischemia and reperfusion model. The results revealed that the cardio-protective effects of Heliox pre-conditioning in young rats were due to two additional mechanisms: mitochondrial uncoupling and Ca^2+^ sensitive potassium channel activation [39].

Helium protects healthy myocardium against ischemia & reperfusion injury by early and late preconditioning (EPC,LPC) and postconditioning (PostC). In 2012, Weber et. al found that triple intervention of EPC, LPC, and PostC significantly reduced infarct sizes in spontaneously hypertensive rats [40].

### Application of helium in neurology

The lighter inert gases neon and helium are not anesthetics at least up to the highest pressures that can be tolerated before the confounding effects of high-pressure neurological syndrome become pronounced [14,41]. At these high pressures (~ 100 atm), the manifestations of high-pressure nervous syndrome include hyper excitability, tremors, and convulsions which would act to oppose any sedative or anesthetic effect [42,43].

Evidence that xenon has neuroprotective properties has prompted researchers to explore whether or not other inert gases have this potential. Only a few studies have investigated the effects of Heliox treatment or pre-conditioning in the nervous system, and the mechanism has yet to be properly elucidated. Pan et. al compared the difference in treatment with hyperoxia and Heliox during the ischemia &reperfusion process in the brain [44]. They found that Heliox was superior to hyperoxia in both in infarct volume reductions and improvements in neurological deficits.

In a similar study, Coburn et. al found that in an *in vitro* model of traumatic brain injury, treatment with helium at elevated pressures had neuroprotective effects [45]. In a different *in vitro* study of cultured neurons however, Rivzi et. al reported that normobaric helium was detrimental to neuron survival after hypoxia [46], and human tubular kidney cells [47]. The reason for the differing results has not been made clear.

In another study, rats treated with Helium below body temperature subjected to middle cerebral artery occlusion (MCAO) had decreased infarct size and improved neurological outcome. In rats treated with Helium at 33°C, the neuroprotective effect of Helium was abolished. The authors contributed the beneficial effect of helium to hypothermia caused by the high thermal conductivity of helium compared with air [4]. While many pharmacological targets have been discovered for xenon, no targets have yet been discovered for helium, [48] opening up many future investigational opportunities.

Remarkably, an *in vitro* study of Schwann cells isolated from sciatic nerves of 4–5 day old rats; researchers found that irradiating the cells with a Helium-neon laser caused proliferation of the cells in a dose dependent manner [49]. This application is promising in regards to neuron restoration post injury.

### Helium gas in surgery

In 2003, Brackman et. al found that helium pneumoperitoneum ameliorates hypercarbia and acidosis associated with carbon dioxide insufflation during laparoscopic gastric bypass in pigs [50].

Laparoscopic surgery is now a widely performed in treating various abdominal diseases. The procedure requires distending the abdomen via insufflation with carbon dioxide gas to visualize abdominal structures and provide space for the manipulation of medical instruments. Carbon dioxide is absorbed by the peritoneum and alters physiologic parameters, which can complicate surgery: mainly changes to the heart and lungs (cardiopulmonary changes). Cheng et. al performed a meta-analysis of all the studies using other medical gases, nitrous oxide and helium, in creating the pneumoperitoneum required for performing abdominal laparoscopic surgery. Their results concluded that there were fewer cardiopulmonary changes with helium than with carbon dioxide [51].

Helium has been found to be a safe alternative as an insufflant in high-risk patients undergoing laparoscopic renal surgery. Researchers cite that patients who benefit most are those with difficulty in clearing CO_2_ gas from their bloodstream, such as patients with comorbid conditions like COPD, congestive heart failure, chronic hypoxia from an intrapulmonary shunt, malignant hyperthermia, and chronic hypoxia from multiple pulmonary infarcts [52]. In general surgery, helium is being explored as a promising abdominal insufflant alternative to CO_2_ because in laboratory and clinical trials, helium has not produced the respiratory acidosis commonly associated with insufflation using CO_2_[53]. Furthermore, Waseda et. al found that helium pneumoperitoneum could improve the recovery of postoperative gastrointestinal motility because of the reduction of hypercapnia and acidosis compared with CO_2_ pneumoperitoneum [54].

Helium plasma technology has also found an application in abdominal and laparoscopic surgery. Helium plasma is being used in the thermal coagulation of tissues that clears the bleeding from the surgical field and enhances visualization of bleeding sites [55].

### The future of helium research

Since the discovery of helium, the applications of helium have evolved greatly. Current research shows that helium gas is being studied in cutting edge technologically advanced medical fields such radiology and microscopy.

### Helium in radiology

Because of inherently low ^1^H abundance in the lungs, MRI of the lungs has been more challenging to adequately visualize than other body tissues. Furthermore, air-tissue interfaces in the lung create magnetic field distortions, which further diminishes the lung magnetic resonance ^1^H signal. Respiratory and cardiac motion further deteriorates pulmonary MRI quality [56].

Inhaled, hyperpolarized helium (HP ^3^He) overcomes the low proton density in both normal and diseased lungs. Polarization of largely achieved using the spin exchange optical pumping method (SEOP) [57-59]. The nuclear polarization of the unpaired nuclear proton increases up to five orders of magnitude compared to a modest linear increase with field strength using thermal polarization [60-62]. The application of hyperpolarized Xenon (HP ^129^Xe) MRI has lagged behind HP ^3^He MRI methods mostly because ^129^Xenon is more challenging to polarize [59].

Using the SEOP method, the helium gas is polarized overnight (12–14 hours) and inhaled by subjects from a bag mixed with medical nitrogen for immediate breath-hold imaging (8–16 sec). The method is safe, requires no ionizing radiation dose, and can be repeatedly inhaled facilitating longitudinal [63,64], interventional [65], and pediatric exams [66]. HP ^3^He MRI can provide additional information regarding lung oxygenation that was not possible with traditional high-resolution computed tomography (HRCT) or MRI.

### Helium in microscopy

Helium ion scanning microscopy (HIM) is a novel imaging technology with the capability of providing sub-nanometer resolution images of uncoated biologic tissues. Taking advantage of helium ion microscopy, Rice et. al were able to explore the epithelium of the rat kidney with unsurpassed image quality and detail [67]. With the helium ion system, fine details such as membrane texture and membranous nanoprojections on glomerular podocytes were visualized.

## Conclusion

**Figure 4:**
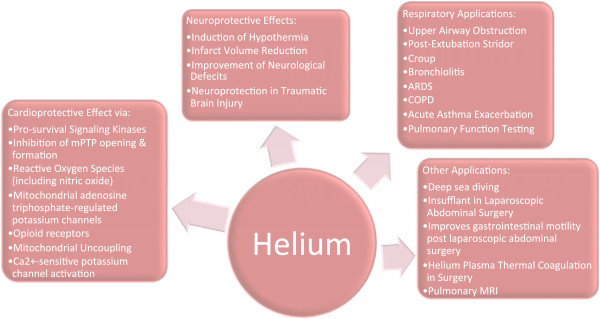
A summary of the many known effects and applications of helium gas in medicine.

In conclusion, the many applications of helium gas in medicine are due to its unique physical and chemical properties including its low solubility, high thermal conductivity, and low density. Helium has been most studied as a possible adjunct to respiratory therapy. Studies investigating helium gas regarding protection of the myocardium after ischemia has elucidated many mechanisms for a gas once thought to be biologically inert. With regards to neuroprotection, more studies are required to elucidate the neuroprotective mechanism that Helium gas has on neurons and to resolve some controversies in the literature. In general surgery, applying helium during laparoscopic surgery has beneficial effects not offered traditionally by carbon dioxide. Helium has exciting new applications in medicine with regards to MRI imaging of the lungs, and microscopic imaging (Figure 4).

## Abbreviations

ARDS: Acute respiratory distress syndrome; COPD: Chronic obstructive pulmonary disease; EPC: Early preconditioning; Heliox: Helium-oxygen; HIM: Helium ion microscopy; HP 3He: Hyperpolarized helium; HP 129Xe: Hyperpolarized xenon; LAD: Left anterior descending; LPC: Late preconditioning; MCAO: Middle cerebral artery occlusion; MRI: Magnetic resonance imaging; PostC: Post-conditioning; RSV: Respiratory syncytial virus; SEOP: Spin exchange optical pumping; WOB: Work of breathing.

## Competing interests

The authors declare that they have no competing interests.

## Authors’ contributions

CJB-Role included reviewing manuscripts, review design, manuscript preparation, and manuscript editing. JZ-Role included review design and manuscript proof reading. Both authors read and approved the final manuscript.
